# Human hepatitis D virus-specific T cell epitopes

**DOI:** 10.1016/j.jhepr.2021.100294

**Published:** 2021-04-23

**Authors:** Matin Kohsar, Johanna Landahl, Christoph Neumann-Haefelin, Julian Schulze zur Wiesch

**Affiliations:** 1 I. Medical Department (Gastroenterology with the Sections Infectiology and Tropical Medicine), University Medical Center Hamburg-Eppendorf, Hamburg, Germany; 2 Department of Medicine II (Gastroenterology, Hepatology, Endocrinology, and Infectious Diseases), Freiburg University Medical Center, Faculty of Medicine, University of Freiburg, Freiburg, Germany; 3 AKK Altonaer Kinderkrankenhaus gGmbH, Bleickenallee 38, 22763 Hamburg, Germany; 4 German Center for Infection Disease (DZIF), University Medical Center Hamburg-Eppendorf, 20246 Hamburg, Germany

**Keywords:** Hepatitis Delta, HBV, HDV, T cell, CD8+, CD4+, epitope, viral escape, aa, amino acid(s), ADAR1, adenosine deaminases acting on RNA, ALT, alanine aminotransferase, AST, aspartate aminotransferase, cccDNA, covalently closed circular DNA, ELISpot, enzyme-linked immune spot assay, HDAg, hepatitis delta antigen, ICS, intracellular cytokine staining, IFN-, interferon-, L-HDAg, large hepatitis delta antigen, MAIT, mucosa-associated invariant T cells, NK cells, natural killer cells, NTCP, sodium taurocholate co-transporting polypeptide, PBMCs, peripheral blood mononuclear cells, PD-1, programmed cell death protein 1, Peg-IFN-α, pegylated interferon alpha, PTM, post-translational modification, S-HDAg, small hepatitis delta antigen, TCF, T cell-specific transcription factor, Th1, T helper 1, TNFα, tumour necrosis factor-α

## Abstract

HDV is a small, defective RNA virus that requires the HBsAg of HBV for its assembly, release, and transmission. Chronic HBV/HDV infection often has a severe clinical outcome and is difficult to treat. The important role of a robust virus-specific T cell response for natural viral control has been established for many other chronic viral infections, but the exact role of the T cell response in the control and progression of chronic HDV infection is far less clear. Several recent studies have characterised HDV-specific CD4+ and CD8+ T cell responses on a peptide level. This review comprehensively summarises all HDV-specific T cell epitopes described to date and describes our current knowledge of the role of T cells in HDV infection. While we now have better tools to study the adaptive anti-HDV-specific T cell response, further efforts are needed to define the HLA restriction of additional HDV-specific T cell epitopes, establish additional HDV-specific MHC tetramers, understand the degree of cross HDV genotype reactivity of individual epitopes and understand the correlation of the HBV- and HDV-specific T cell response, as well as the breadth and specificity of the intrahepatic HDV-specific T cell response.

**Key points**

• HDV causes severe hepatitis, often leading to hepatic complications and liver-related death; it is a major public health concern affecting 12 million patients worldwide, with few treatment options.
• The virology and immunology of HDV infection, which is intricately connected with the concomitant HBV infection, is still not completely understood; it is only recently that several studies characterising the T cell response in patients with HDV have been published.
• This review summarises our current knowledge on the virology and immunology of HDV infection, with a focus on the HDV-specific T cell response.
• A comprehensive database of all HDV-specific CD4+ and CD8+ T cell epitopes published to date is presented.
• Detailed functional and phenotypic studies on the peripheral and intrahepatic HDV-specific T cell response during future clinical trials are needed to understand the T cell corelates of HDV control.

## Introduction

Apart from some initial immunological HDV studies in the 1990s, it was only more recently that several immunological research groups further characterised the HDV-specific T cell response using state of the art methods. In this comprehensive review, we summarise these studies and list all HDV-specific T cell epitopes identified in humans so far, with a focus on their potential significance as well as unresolved knowledge gaps. Detailed knowledge of the HDV-specific T cell epitope repertoire is needed to guide therapeutic vaccine design and to improve immune monitoring in future clinical trials.

## Epidemiology

The global prevalence of HDV is estimated at 12 million individuals,^1^ while others have calculated that it affects 32–61 million,^2^^,^^3^ or even 62–72 million individuals.^4^ HDV is highly endemic in Africa, the Amazon Basin, Eastern and Mediterranean Europe, the Middle East, and parts of Asia,^4^ mostly coinciding with high numbers of chronic HBV infections in these areas. HDV genotype 1 has global prevalence, while genotypes 2-8 show distinct regional patterns.^5^ Genotypes 2 and 4 predominantly cause milder disease, while the South American genotype 3 causes more severe hepatitis.^6^ Genotypes 5-8 are mostly diagnosed in patients of African origin and have also been linked to milder disease,^7^ although the latter is a matter of debate.^8^ Untreated chronic HBV/HDV infection causes severe liver disease in many cases, with 50% of patients developing cirrhosis within 5-10 years.^6^ Whether HBV/HDV infection is associated with an increased risk of HCC *per se*,^9^ or whether this only occurs secondary to cirrhosis,^10^ is controversial.

HDV remains a high-priority public health concern^11^ for 3 main reasons: increasing immigration from HDV endemic areas towards the US and Northern Europe; high endemicity of HDV in low-income countries; ongoing outbreaks of HDV.^5^^,^^6^ HDV elimination thus depends on both specific therapies as well as preventive HBV vaccination.

## HDV virology

HDV is an exceptionally small virus^12^ and considered defective due to its dependency on the HBV-derived HBsAg to form infectious virions. HDV cell entry is dependent on the interactions between HBsAg/heparan sulfate proteoglycans and sodium taurocholate co-transporting polypeptide (NTCP).^13^ HDV might also use envelope proteins of other viruses for transmission,^14^^,^^15^ however, the clinical significance is unknown.^16^ HDV contains a circular single-stranded RNA genome of 1.7 kb, encoding a single 214-amino acid (aa) peptide,^5^ the hepatitis delta antigen (HDAg), which exists in 2 variants, the small HDAg (S-HDAg) and the large HDAg (L-HDAg), which has 19 additional C-terminal aa.^12^ Approximately 200 of these molecules are included per virion,^5^^,^^17^ in the form of nucleosome-like ribonucleoproteins consisting of the HDAgs and viral RNA, which have essential roles in viral replication.^18^^,^^19^

**Fig. 1:**
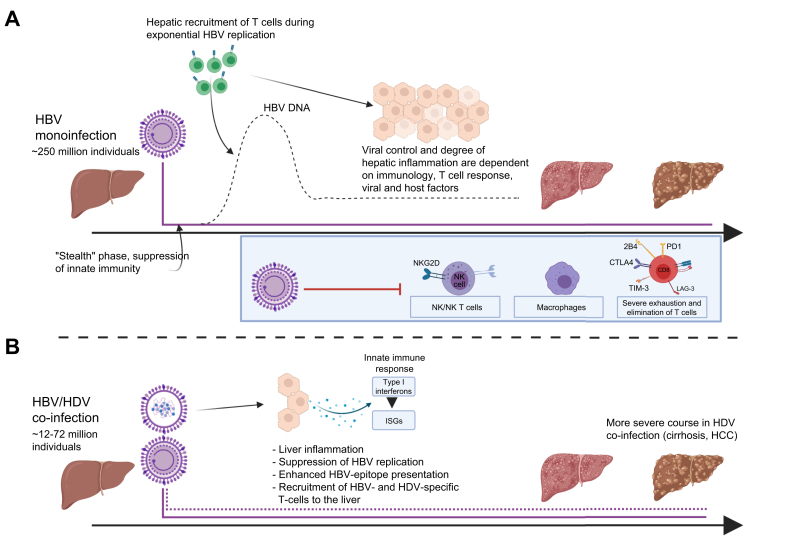
Immunological course of HBV monoinfection *vs.* HBV/HDV coinfection. Note that chronic HBV inhibits various pathways of innate immunity and leads to different degrees of T cell exhaustion and deletion. Clearance of HBV is largely dependent on effective CD4+ and CD8+ T cell responses, as well as innate immune response, viral and host factors.^117^ Contrary, HDV activates pathways of innate immunity, thereby increasing type-I interferon (β and λ) responses and suppressing HBV replication. HBV epitope presentation and hepatotropic T cell recruitment is enhanced. Which immune responses are primarily required for HDV clearance is not well understood.^12^^,^^74^^,^^76^ ISG, interferon-stimulated genes; NKG2D, natural killer group 2 member D. Figure created with Biorender.com.

HDV RNA is replicated and transcribed to host-like^20^^,^^21^ mRNA in a double rolling circle process by host DNA-dependent RNA polymerases, mainly RNA pol II, but possibly also RNA pol I and III.^22^^,^^23^ The small genome and the replication mode are unique among animal RNA viruses and more typical for plant viroids/virusoids.^24^^,^^25^ Other than these, HDV hijacks host enzymes and even forces a template shift from host DNA to viral RNA,^25^^,^^26^ probably using a histone H3 mimicry strategy.^19^

L-HDAg is translated after ADAR1 (adenosine deaminases acting on RNA 1)-mediated editing of a stop codon at the amber/w site (adenosine 1012) at a portion of the *antigenomic* RNA, effectively elongating the open reading frame by 19–20 aa.^12^^,^^27^^,^^28^ Thus, S-HDAg is the first of the 2 peptides and facilitates replication, while L-HDAg is translated at a later stage and inhibits replication to promote virion assembly.^29^

Post-translational modifications (PTMs) are essential for the function of HDAg.^30^ Namely serine-2, -123 and -177 are phosphorylated post-translationally, arginine-13 is methylated, and lysine-72 is acetylated. Cysteine-211, only found in the L-HDAg, is modified by isoprenylation/farnesylation. It has been reported that the acetylation of lysine-72 is required for the subcellular localisation of HDAg and RNA replication. Other important PTMs in this regard include the methylation of arginine-13 and the phosphorylation of serine-177 and -123. The farnesylation of cysteine-211 is required for virus assembly^13^^,^^30^^,^^31^ and to inhibit replication.^32^

Other functional domains include a coiled-coil domain that is important for self-dimerisation,^23^ a domain that determines the nuclear localisation of HDAg^33^ and the unique carboxyterminal region of L-HDAg with the nucleolar export signal.^34^ RNA-binding arginine-rich motifs of HDAg have been described, still, oligomerisation seems more important for the activating and inhibitory effects of S- and L-HDAg.^25^^,^^35^

## Heterogeneity and viral evolution

HDV shows great genetic variance, a broad range of viral quasispecies exist within the same infected individual.^5^ The intragenotypic genetic variability of HDV genotype 1 is estimated to be 11.3–14.3%^36^ and, recently, a subclassification of genotypes was proposed.^37^

Like DNA polymerases, DNA-dependent RNA polymerase II is reported to have kinetic proofreading abilities, however, the template switch to RNA might cause a higher error rate.^38^ Additionally, stray ADAR1-mediated RNA editing might also contribute to sequence heterogeneity.^39^

The substitution rates range from 3.0∗10^-2^ to 3.0∗10^-3^ for the whole genome and 9.5∗10^-3^ to 1.2∗10^-3^ substitutions per site per year for the HDAg open reading frame (determined by next-generation sequencing),^13^ decaying over time towards a steady state.^40^ High evolution rates correlate with clinical flares, and evolution rates are only higher than other RNA viruses at the beginning of the infection, during adaptation to the host.^41^ Non-synonymous mutations happen relatively more often, likely as a result of selection of variants capable of immune escape.^42^

PTM sites and ribozymes seem rather conserved, while 10.6% of codons are under diversifying positive selection.^43^

A reduced *in vitro* sensitivity of HDV to interferon (IFN)-α during treatment has been reported,^44^ likely due to the selection of genetic variants that replicate despite IFN. Possibly, HDV is activating the IFN pathways itself to suppress HBV replication and increase RNA editing by the IFN-induced enzymes ADAR1^45^^,^^46^ and apolipoprotein B mRNA editing enzyme (APOBEC).^13^^,^^44^

In conclusion, HDV shows higher initial mutation rates than other hepatotropic viruses, while a few conserved genomic regions are described. Mutations and quasispecies may contribute to immune escape and treatment failure.

## HDV therapy

Until recently, the only recommended treatment option for chronically HBV/HDV-infected patients was a long-term (48 weeks) therapy with pegylated-IFN-α (peg-IFN-α).^47^ Only few patients respond to treatment and late relapses occur in almost half of responding patients after achieving a ‘sustained’ virological response.^48^ Only 11% of patients maintain a virological response after IFN-based regimens and late relapses after therapy discontinuation are not uncommon.^49^ Elongation of IFN therapy to 96 weeks is possible with acceptable safety in up to 80% of patients, leading to longer on-treatment suppression of HDV replication and amelioration of fibrosis.^50^ However, relapse rates are still high (around one-third of responders) and HBsAg clearance is not improved – even by addition of tenofovir disoproxil fumarate^50^ – which is possibly linked to almost undetectably low viraemia.^51^ These findings underscore that, while a sustained virological response by negative HCV PCR 12 weeks after therapy indicates cure from HCV, this concept cannot be extrapolated to HDV, where loss of HBsAg and seroconversion remain the best markers for cure of chronic HDV infection.^49^^,^^52^ Roche has officially stopped the production of peg-IFN-α which will only be available until the end of 2022.

The novel entry inhibitor bulevirtide targets host NTCP and has led to promising results in 2 phase II trials,[53], [54], [55] leading to its conditional marketing authorisation in Europe.^56^^,^^57^ Other novel anti-HDV agents are currently being investigated (reviewed in^53^ and ^58^). Pegylated-IFN-λ showed advantageous tolerability and comparable antiviral activity during 8 weeks of treatment compared to 48 weeks of peg-IFN-α in a randomised, open-label, multicentre study.^53^^,^^59^ Lonafarnib – an orphan drug for the rare genetic disorder progeria – inhibits the host enzyme farnesyltransferase and is being investigated for HDV therapy in multiple combinations.^53^ The HBsAg release inhibitor REP-2139 is another promising candidate for HDV treatment.^60^ So far, although paradigm-shifting, none of these novel therapeutic approaches is recommended in international treatment guidelines.^47^^,^^61^

## Immunology of HDV infection

Humans are the only natural host of HDV and only chimpanzees^62^ and some non-primates such as Tupaia bengaleri^63^ can be infected with (human) HBV and co-/superinfected with HDV. Other mammals used for prospective studies on HDV are woodchucks, woolly monkeys, and bats.^17^ HDV-transgenic mice were used to demonstrate that HDV hepatotropism is only due to the entry restriction by HBsAg.^17^ HDAg/HBsAg-transgenic mice did not develop liver disease.^64^ *In vitro*, in transient transfection, there was no interference with the cell cycle or apoptosis, whereas in dividing cells a slight growth disadvantage could be observed.^65^ Accordingly, HDV may – to some degree – be cytopathic itself and drive histopathologic liver damage together with the immune response.^66^ In humanised uPA/SCID/beige mice, HDV monoinfection can persist intrahepatically for at least 6 weeks without HBsAg, while maintaining infectivity and the ability to convert to a productive co-infection after rescue by HBV infection.^67^

**Fig. 2:**
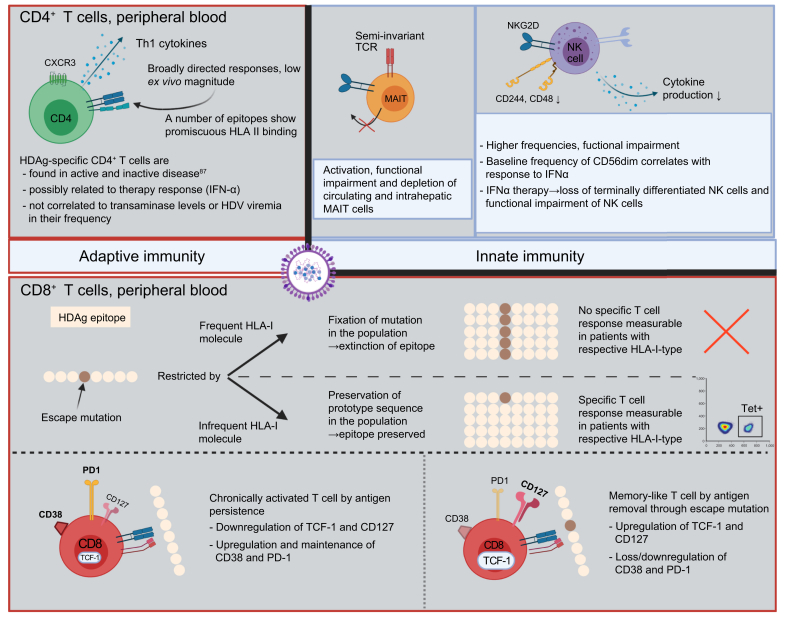
Key immunological findings in chronic HDV. CXCR3, CXC-motif chemokine receptor 3; MAIT, mucosal associated invariant T cell; TCR, T cell receptor; TCF-1, T cell factor 1; PD-1, programmed cell death protein 1; Tet, HLA-I tetramer (loaded with HDAg-derived peptide). Based on [82], [83], [84],[86], [87], [88], [89],^91^,^92^. Figure created with Biorender.com

HDV and HBV interact in multiple ways. Although HDV is able to replicate without active HBV replication,^67^ HBsAg is needed for HDV to form infectious particles. Usually, HDV predominates over HBV and coinfected patients often only show low HBV viral loads, although this pattern might be reversed in some individuals^68^ or transiently during early treatment phases.^69^

*In vitro*, upon superinfection there is a specific interference between the 2 viruses: HBV DNA, pregenomic RNA and HBeAg decrease while cccDNA and HBsAg stay constant.^70^ HDV infection is associated with a type-I IFN response and upregulation of IFN-induced genes.[70], [71], [72], [73] Upregulation of IFN-induced genes not only increases the HDV mutation rate, but also suppresses HBV replication,^23^ owing to decreased transcription of covalently closed circular DNA (cccDNA, without a decline in cccDNA abundance) as a result of IFN-α-induced epigenetic changes to the cccDNA.^74^^,^^75^ Notably, IFN partially suppresses HDV replication under certain circumstances (reviewed in detail^12^). HDV also enhances HBV epitope presentation, which could be one of the causes of the more severe liver pathology in HBV/HDV coinfection (Fig. 1).^76^

Few studies examined humoral immune responses to HDV. Anti-HDV antibodies are commonly generated,^77^ but probably unable to neutralise HDV.^66^^,^^78^ Anti-HDV IgM was traditionally used as clinical marker of disease activity before the establishment of standard pan-genotypic PCR assays.^79^^,^^80^

Chronically HDV-superinfected patients have higher serum type 1 to type 2 cytokine ratios, while HBV-monoinfected patients show elevated levels of both type 1 (tumour necrosis factor-α [TNFα], interleukin [IL]12, C-X-C motif chemokine ligand 9, IFN-γ) and type 2 (IL4, IL13, C-C motif chemokine ligand 26) cytokines.^81^ This predominance of type 1 responses, which mainly elicit cellular immune cascades, might explain the more aggressive course of disease in the case of HDV superinfection.^81^ Further, it has been shown that HDV strongly activates an IFN-β/λ response mainly through the pattern recognition receptor MDA5 (melanoma differentiation-associated protein 5) and that HDV can replicate *in vivo* despite this “interferon-activated state”.^44^

Functionally impaired CD56^bright^ natural killer (NK) cells accumulate in viral hepatitis regardless of the virus itself (Fig. 2).^82^ The highest total frequencies of NK cells and CD56^dim^ NK cells and the highest amounts of IFN-γ and TNF-α were found for HDV. However, phenotype and functional alterations were attributed primarily to the severity of infection rather than the virus itself.^82^ A higher frequency of CD56^dim^ NK cells in HDV-infected patients is associated with better outcome after IFN-α treatment.^83^ IFN-α treatment seems to deplete terminally differentiated NK cells and cause functional impairment of NK cells.^83^

Intrahepatic and peripheral frequencies of mucosa-associated invariant T (MAIT) cells are reduced in patients with chronic HDV compared to healthy individuals and patients with HBV monoinfection of similar age.^84^ MAIT cells are also functionally impaired and exhibit an activated and exhausted compound phenotype of CD38^hi^PD-1^hi^CD28^lo^CD127^lo^PLZF^lo^Eomes^lo^Helios^lo^ .^84^

Similar to chronic HBV and HCV infection,^85^ it is assumed that specific T cell responses are needed for clearance of HDV and, at the same time, are drivers of histopathologic liver damage.^78^ There is great variability in the observed frequencies of HDV-specific T cell responses reported in the literature. Grabowski *et al.* describe HDV-specific cytokine responses after stimulation of peripheral blood mononuclear cells (PBMCs) with a peptide pool spanning the whole HDAg in 94% (16/17) of patients before the start of IFN treatment.^86^ Nisini *et al.* detected HDV-specific CD4+ T cell proliferation in 27% of patients after whole-antigen stimulation (8/30),^87^ whereas Landahl *et al.* detected CD4+ or CD8+ T cell responses in 53% of patients (17/32) by intracellular cytokine staining after *in vitro* expansion (ICS).^88^ Kefalakes *et al.* report an HDV-specific T cell response rate of 71% by IFN-γ ICS in a sub-cohort of 17 lonafarnib/ritonavir-treated patients after treatment discontinuation.^89^ *Ex vivo* frequencies of epitope-specific CD8+ T cells were either undetectable,^90^^,^^91^ detectable at very low frequencies after enrichment^92^ or reported at 0.013% of CD3+CD4- T cells,^89^ depending on the epitopes and multimers used.

Even less is known about the phenotype and the clinical correlate of the detection of a broad HDV-specific T cell response. Specific T cell responses have been linked to “inactive disease” – defined as normal alanine aminotransferase (ALT) levels for 1 year and negative anti-HDV IgM.^87^ A higher frequency of HDV-specific cytokine responses and a restoration of transiently diminished specific cytokine responses after peg-IFN-α treatment coincided with therapeutic response in the HIDIT-1 trial. However, this correlation between HDV-specific IFN-γ levels, ALT levels and HDV RNA was not significant.^86^ Correlations between elevated serum IL2 and IL12 levels and response to IFN treatment^93^ suggest that a T helper-1 (Th1)-polarised cellular immune response might be associated with viral clearance.

In contrast, Landahl *et al.* observed broad low-level HDV-specific T cell responses that did not correlate with HDV viral load, level of transaminases and presence or absence of HDV viraemia. There was also no difference between responses in spontaneous resolvers, treatment-induced PCR-negative patients and chronically viraemic patients, but there was a negative correlation between HBV viral load and number of responses.^88^ Interestingly, a correlation between activated HDV-specific T cells and aspartate aminotransferase (AST) levels was reported,^89^ suggesting that CD8+ T cells may contribute to liver damage in HDV infection. Furthermore, higher frequencies of IFN-producing CD8+ T cells were associated with lower viremia 4 weeks post treatment, emphasising their role in viral clearance.^89^ All in all, the exact interplay between specific T cell responses and treatment outcome or disease course remains unclear.

Cytokine secretion analyses suggest that HDV-specific CD4+ T cell clones belong either to Th1 or Th0 subsets with some cytotoxic capabilities.^87^ HDV-specific CD8+ T cells were described as non-terminally exhausted memory cells, with a lesser degree of CD8+ T cell exhaustion than in Epstein-Barr virus infection^89^^,^^91^ and as memory-like and “chronically activated, but not terminally differentiated”.^89^ Some of the epitope-specific memory-like CD8+ T cells were predominantly T cell-specific transcription factor 1 (TCF1)-positive, which was attributed to viral escape mutations resulting in loss of antigen recognition and a relative expansion of TCF1-positive memory-like cells in comparison to relatively diminishing amounts of exhausted effector cells.^89^^,^^92^ Interestingly, a quite similar CD127+PD1+TCF1+ population of epitope-specific CD8+ T cells, sharing characteristics of T cell memory and exhaustion, has been described in HCV.^94^ In HIV, TCF-1 expression maintains stem-cell like properties of virus-specific CD8+ positive T cells, thereby preventing exhaustion and is linked to the elite controller status.^95^ Significantly higher frequencies of perforin-positive CD4+ T cells could be observed in chronically HDV-infected patients compared to chronically HCV- or HBV-infected patients and correlated with elevated AST and decreased platelet count in one study.^96^ However, the antigen specificity of these perforin+CD4+ T cells was not assessed.

### HDV-specific CD4+ T cell epitopes

In HCV infection, the detection of strong and long-lasting epitope-specific CD4+ T cells correlates with spontaneous viral clearance of acute HCV, while these responses are nearly absent in patients with chronic HCV.[97], [98], [99], [100] Significantly less is known about the role of CD4+ T cell responses in HBV monoinfection or HDV infection.

**Fig. 3:**
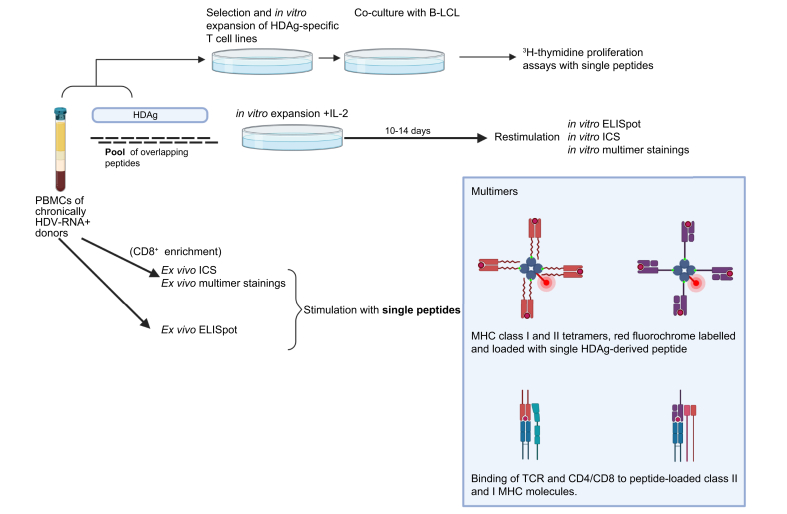
Simplified overview of commonly used methods of epitope detection/mapping. B-LCL, B-lymphoblastoid cell line; ELISpot, enzyme-linked immune spot assay; ICS, intracellular cytokine staining; IL-2, interleukin-2. Based on [87], [88], [89], [90], [91], [92]. Figure created with Biorender.com

However, some studies have longitudinally characterised the HDV CD4+ T cell response during the primary HBV/HDV coinfection or acute HDV superinfection of HBsAg carriers using standardised CD4+ T cell assays (Fig. 3). So far, responses to 18 different HDAg-specific CD4+ T cell epitope specificities have been described in 2 studies,^87^^,^^88^ 4 of these epitopes were detected in more than 1 tested individual. Landahl *et al.* identified 14 epitopes by *in vitro* enzyme-linked immune spot assay (ELISpot) using overlapping 20mer peptides spanning the whole L-HDAg, followed by ICS for IFN-γ in patients with positive ELISpots results.^88^ Nearly the whole HDAg was immunogenic for CD4+ responses, however, the hotspots were located towards the N-terminus. One of these, aa71-90, contains the nuclear localisation signal. The CD4+ T cell responses against the 2 N-terminal epitopes aa21-40 and 41-60 were also confirmed in a patient with acute HDV infection using direct *ex vivo* ELISpot assays.^88^

For assessment of the HLA restriction of the epitopes, Landahl *et al.* combined *in silico* predictions, HLA binding assays and HLA typing of responding patients to suggest likely HLA restrictions. Epitopes aa11-30 and 41-60 seem to bind to multiple HLA molecules in a rather promiscuous fashion. Truncating experiments suggest restriction by DRB1∗11:01 as described by Nisini *et al.* for an overlapping peptide (see below).

Interestingly, no difference was found in the detection rate, breadth or magnitude of the overall low-level CD4+ HDV-specific T cell responses between PCR-positive and negative patients.^88^

Nisini *et al.* tested pools of overlapping 16mer peptides spanning the whole L-HDAg on HDV-specific CD4+ T cell lines derived from 3 chronically HDV-infected patients with inactive disease (defined as normal ALT blood levels and undetectable anti-HDV IgM).^87^ Epitope-specific T cell proliferation was measured in a 3H-thymidine proliferation assay. The patients were preselected from a larger cohort of 30 patients based on their responsiveness to whole HDAg.

Four epitopes were identified by pool stimulations. Peptides were synthesised according to a genotype 1 sequence. However, the exact sequences of the tested peptides were not provided in the publication.^87^ HLA restrictions were studied by blocking experiments with anti-DR, anti-DP, and anti-DQ monoclonal antibodies. A B-lymphoblastoid cell line containing known haplotypes was then co-cultured with HDV-specific CD4+ T cell clones to assess their binding to certain HLA molecules.

The epitopes seem to be presented in conjunction with multiple MHC class II molecules, mostly DRB1 subtypes. Nisini *et al.* also described 13 additional epitopes in the study (with response rates ranging from 1 out of 7 to 3 out of 7 patients) by 3H thymidine proliferation of whole PBMCs derived from patients who responded to stimulation by whole HDAg. Given the stimulation mode, these are most likely CD4+ T cell epitopes, however, this has not been experimentally confirmed.^87^

**Fig. 4:**
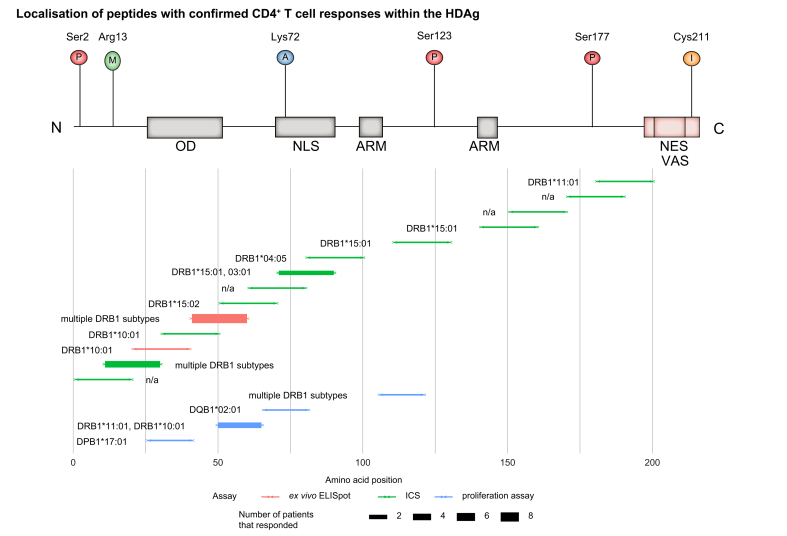
Schematic overview of peptides eliciting CD4+ T cell responses in relation to the L-HDAg. Bar thickness of lower plot represents number of responding patients, colour represents type of assay performed. Upper pictogram shows PTM sites and functional domains of HDAg, based on 17. ICS, intracellular cytokine staining; L-HDAg, large hepatitis delta antigen.

A schematic overview of the CD4+ T cell epitopes and their localisation within the HDAg is provided in Fig. 4.

### HDV-specific CD8+ T cell epitopes

It is generally thought that HBV-specific CD8+ cells are the main effector cells responsible for viral clearance during acute HBV infection.^101^ The role of CD8+ T cells in the different disease courses of patients with chronic HBV is less clear. CD8+ T cells cause liver injury and promote disease pathogenesis. A key event in the persistence of HBV is the exhaustion of virus-specific CD8+ T cells – indeed, diminished frequencies of functionally impaired HBV-specific CD8+ T cells expressing inhibitory receptors have been described. Immune checkpoint inhibition could restore antiviral CD8+ T cells responses.^102^

The role of the HDV-specific CD8+ T cell response in HDV resolution and pathogenesis remains unclear and most of the data are derived from animal models.^66^^,^^103^^,^^104^ Further, definition of the specificities of the anti-HDAg-specific T cells is an essential step towards understanding the heterogenous disease courses of HDV infection, non-responders, and paving the way for an immunotherapeutic approach.

So far, T cell responses directed against 18 HDV-specific CD8+ T cell epitopes have been identified in 5 different studies, with partial overlap between the studied peptides.[88], [89], [90], [91], [92]

Huang *et al.* described the 2 CD8+ T cell epitopes that were restricted by HLA-A∗02:01 as predicted *in silico*. Out of 4 HLA-A∗02-positive patients, responses were detected in the 2 PCR-negative patients with normal ALT levels by ELISpot and HLA-A∗02:01 tetramer staining.^90^ However, these epitopes were not detectable in 4 European HLA-A∗02-positive patients by ICS,^91^ the overall response rate therefore being 2/8 for each epitope in a total of 2 studies.^90^^,^^91^ Possibly, different HLA-A∗02 subtypes in Asia and Europe may play a role in these discordant results.

Karimzadeh *et al.* analysed HLA-B∗27-restricted HDV-specific CD8+ T cell responses based on HLA-B∗27-associated sequence polymorphisms (indicating viral escape mutations within a putative HDV-specific CD8+ T cell epitope, see below). By IFNγ ICS, 2 epitopes were confirmed: aa99-108 and aa103/104-112. Three patients with resolved HDV infection responded to these 2 epitopes (1 patient for each epitope plus 1 patient who was only tested for the overlapping peptide containing both epitopes).

In 2019, Karimzadeh *et al.* expanded their viral sequence-based approach to all HLA class I alleles and described 5 additional HDV-specific CD8+ T cell epitopes by IFN-γ ICS of *in vitro* expanded CD8+ T cells from HLA-matched patients. Out of these HLA-B restricted novel epitopes, aa170-179 was additionally confirmed by direct *ex vivo* analysis after HLA-B∗15:01 tetramer enrichment.

Kefalakes *et al.* 2019 used overlapping peptides spanning the whole L-HDAg to detect HDV-specific CD8+ T cells. They found responses against a total of 6 HDV-specific CD8+ T cell epitopes, including the aforementioned HLA-B∗27 epitope aa104-112^91^ (with an arginine at the C-terminus), the HLA-B∗18 epitope aa46 - 54^92^ (additionally restricted by HLA-B∗44:02 and B∗44:03), and 4 additional novel epitopes.^89^ Most of these epitopes were further confirmed by direct *ex vivo* multimer staining. Response rates for these epitopes varied, however, responses clustered against epitopes located at the C-terminus of HDAg, which is unique to L-DHAg, with up to 8/17 patients responding to individual overlapping peptides, irrespective of the individual HLA types.

In addition to the identification and characterisation of HDV-specific CD4+ T cell epitopes (see above), Landahl *et al.* also found responses against 5 HDV-specific CD8+ T cell epitopes. However, fine-mapping and HLA class I restriction experiments were not performed in this study which focused on HDV-specific CD4+ T cell responses. *In silico* prediction, as well as HLA typing of responding patients, indicated that these responses were most likely restricted by B∗35:01, B∗51:01 and B∗53:01, indicating that the optimal epitope(s) may be identical to the 3 epitopes aa191–196, aa192–200, and aa194–202 identified by Kefalakes *et al.* in this viral region.^89^

**Fig. 5:**
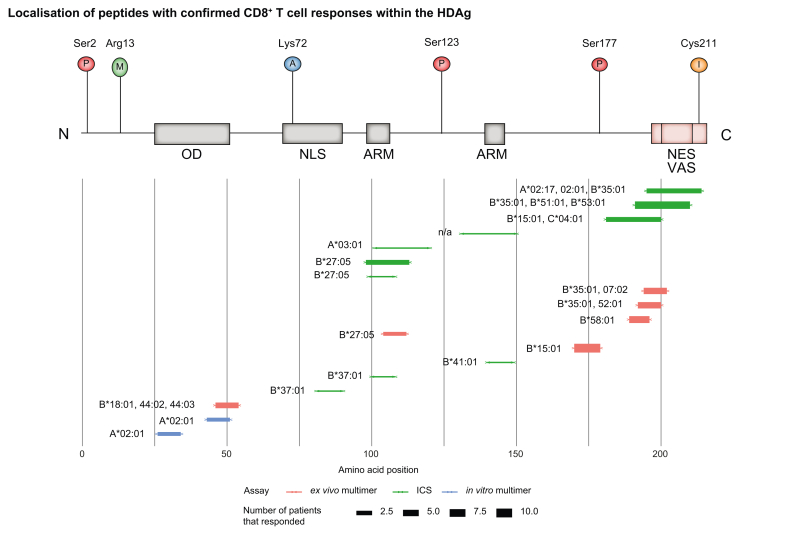
Schematic overview of peptides eliciting CD8+ T cell responses in relation to the L-HDAg. Bar thickness of lower plot represents number of responding patients, colour represents type of assay performed. Upper pictogram shows PTM sites and functional domains of HDAg, based on 17. ICS, intracellular cytokine staining; L-HDAg, large hepatitis delta antigen; PTM, post-translation modification.

Aa101-120 contains the B∗27 epitope aa104-112,^89^^,^^91^ interestingly, the responding patient in ^88^ was HLA-B∗27 negative. Other epitopes also overlap with those identified by the more CD8+ focused studies by Karimzadeh *et al.* and Kefalakes *et al.* (see Table 1 and Fig. 5).

**Table 1:** **Comprehensive overview of all described CD8**^**+**^**and CD4**^**+**^**T cell epitopes of the HDAg**.

Position	Sequence	Ref.	Response rate	Best assay	Assay details	HLA molecule	HLA assay	*In silico predic*tion tool	Comments
**CD8**									
46-54	DENPWLGNI	89,92	4/24	*Ex vivo multim*er	*In vitro* ICS (IFNγ) release^92^; *ex vivo* tetramer ICS^89^	B∗18:01 (multimers); B∗44:02, B∗44:03 (HLA binding)	*In silico,* HLA-matched patients in ICS	ANN, netMHCpan (IEDB; SYFPEITHI; BIMAS	HLA B∗18:01 by multimers, others by binding assays
81-90	VDSGPRKRPL	92	1/1	ICS	*In vitro* ICS (IFNγ)	B∗37:01	*In silico,* HLA-matched patients in ICS	ANN, netMHCpan (IEDB; SYFPEITHI; BIMAS	
100-108	QDHRRRKAL	92	1/1	ICS	*In vitro* ICS (IFNγ)	B∗37:01	*In silico,* HLA-matched patients in ICS	ANN, netMHCpan (IEDB; SYFPEITHI; BIMAS	
140-149	RERRVAGPPV	92	1/2	ICS	*In vitro* ICS (IFNγ)	B∗41:01	*In silico,* HLA-matched patients in ICS	ANN, netMHCpan (IEDB; SYFPEITHI; BIMAS	
170-179	SMQGVPESPF	92	10/14	*Ex vivo* multimer	*In vitro* ICS (IFNγ), *ex vivo* tetramer ICS (7/7)	B∗15:01	*Ex vivo* tetramer ICS, *In silico predic*ions	ANN, netMHCpan (IEDB; SYFPEITHI; BIMAS	
192-200	QGFPWDILF	89	5/17	*Ex vivo* multimer	*In vitro* ICS, *ex vivo* dextramer ICS	B∗35:01; B∗52:01	HLA binding assays with radiolabelled HLA class I, dextramer	n.a.	HLA 35:01 is confirmed by multimer; QGFPWDMLF is also recognized and presented by both HLA-B∗ subtypes; QGFPWDLLF is presented by A∗02:05 und B∗52:01; aa193-200 GFPWDILF is presented by B∗35:01
194-202	FPWDILFPA	89	5/17	*Ex vivo* multimer	*In vitro* ICS, *ex vivo dextra*mer ICS	B∗35:01; B∗07:02	HLA binding assays with radiolabelled HLA class I, dextramer	n.a.	Both HLA types by multimer; FPWDMLFPA also presented by both HLA types (binding assays), FPWDLLFPA: A∗02:05, B∗07:02 and B∗35:01 (binding assays)
104-112	RRKALENK/R	89,92	2/17	*Ex vivo* multimer	*In vitro* ICS, *ex vivo* pentamer ICS; ICS IFNγ release after restimulation on day 12	B∗27:05	HLA binding assays with radiolabelled HLA class I, pentamer	ANN, netMHCpan (IEDB; SYFPEITHI; BIMAS	103-112 RRRKALENKK/R is also presented by HLA B∗27 and recognised by 1/7; escape mutation K106M
189-196	RGSQGFPW	89	6/17	*Ex vivo* multimer	*In vitro* ICS, *ex vivo* tetramer ICS	B∗58:01	HLA binding assays with radiolabelled HLA class I, tetramer	n.a.	
99-108	RRDHRRRKAL	91	1/7	ICS	*In vitro* ICS (IFNγ)	B∗27:05/:02	UV-mediated peptide exchange assay	IEDB and SYFPEITHI	Escape mutations R105K and K106M; RQDHRRRKAL, REDHRRRKAL, RKDHRRRKAL are also presented by B∗27:05 and recognised by one patient each
98-113	ERRDHRRRKALE	91	3/8	ICS	*In vitro* ICS (IFNγ)	B∗27:05	*In silico,* HLA *t*yping in responding patients	IEDB and SYFPEITHI	
26-34	KLEDLERDL	90,91	2/8	*In vitro* multimer	Cytotoxicity (mice) and tetramer staining; ELISPOT IFNγ release, tetramer qualitative binding (both after restimulation); *In vitro* ICS^91^	A∗02:01	MHC ligand assay, UV-mediatied peptide exchange assay	SYFPEITHI	
43-51	KLEDENPWL	90,91	2/8	*In vitro* multimer	Cytotoxicity (mice) and tetramer staining; ELISpot IFNγ release, tetramer qualitative binding (both after restimulation); *In vitro* ICS^91^	A∗02:01	MHC ligand assay, UV-mediatied peptide exchange assay	SYFPEITHI	Karimzadeh *et al.*, found no responses in European cohort by ICS (0/4+2/4); only tested in HLA-A∗02:01 patients
191-210	GQGFPWDILFPS	88	7/32	ICS	*In vitro* ICS (IFNγ); ELISpot IFNγ	B∗35:01; B∗51:01; B∗53:01	*In silico,* HLA typing in responding patients	IEDB Consensus tool (ANN+SMM)	
101-120	DHRRRKALENKR	88	1/32	ICS	*In vitro* ICS (IFNγ); ELISpot IFNγ	A∗03:01	*In silico,* HLA typing in responding patients	IEDB Consensus tool (ANN+SMM)	
131-150	KRLTEEDERRER	88	1/32	ICS	*In vitro* ICS (IFNγ); ELISpot IFNγ	A∗02:02P/03:01P; B∗15:01P/41:01; C∗03:04/17:01P	HLA typing in responding patients	IEDB Consensus tool (ANN+SMM)	
181-200	RHGEGLGVRGG	88	3/32	ICS	*In vitro* ICS (IFNγ); ELISpot IFNγ	B∗15:01; C∗04:01	*In silico, HLA t*yping in responding patients	IEDB Consensus tool (ANN+SMM)	
195-214	PWDILFPSDPPF	88	3/32	ICS	*In vitro* ICS (IFNγ); ELISpot IFNγ	A∗02:17/02:01; B∗35:01	*In silico, HLA t*yping in responding patients	IEDB Consensus tool (ANN+SMM)	

**CD4**									
26-41	Data not provided	87	1/3	^3^H thymidine proliferation	Epitope-specific ^3^H thymidine proliferation after cultivation+stim with HDAg and coculture of B-LCL as APCs	DPB1∗17:01	Blocking experiments with MAbs; co-culture with B- LCL of known haplotypes	no *in silico predic*tions used	
50-65	Data not provided	87	3/3	^3^H thymidine proliferation	Epitope-specific ^3^H thymidine proliferation after cultivation+stim with HDAg and coculture of B-LCL as APCs	DRB1∗11:01; DRB1∗10:01	Blocking experiments with MAbs; co-culture with B- LCL of known haplotypes	no *in silico predic*tions used	
66-81	Data not provided	87	1/3	^3^H thymidine proliferation	Epitope-specific ^3^H thymidine proliferation after cultivation+stim with HDAg and coculture of B-LCL as APCs	DQB1∗02:01	Blocking experiments with MAbs; co-culture with B- LCL of known haplotypes	no *in silico predic*tions used	
106-121	Data not provided	87	1/3	^3^H thymidine proliferation	Epitope-specific ^3^H thymidine proliferation after cultivation+stim with HDAg and coculture of B-LCL as APCs	DRB1∗11:01; DRB1∗11:02; DRB1∗12:01; DRB1∗01:01; DRB1∗07:01; DRB1∗14:01; DRB5∗02:02	Blocking experiments with MAbs; co-culture with B- LCL of known haplotypes	no *in silico predic*tions used	
11-30	GGREEILEQWVN	88	4/32	ICS	*In vitro* (IFNγ)	DRB1∗08:02; DRB1∗10:01; DRB1∗14:01; DRB1∗15:01	*In silico* predictions + MHC ligand assay	IEDB Consensus tool (ANN+SMM)	
41-60	IKKLEDENPWLG	88	8/32	*Ex vivo ELISpo*t	*in vitro* ICS (IFNγ); *ex vivo ELISpo*t IFNγ	DRB1∗10:01; DRB1∗11:01; DRB1∗08:02; DRB1∗13:02	*In silico predic*tions+MHC ligand assay	IEDB Consensus tool (ANN+SMM)	Confirmed by *ex vivo ELISpo*t in acutely superinfected patient
1-20	MSRSESKKNRG	88	1/32	ICS	*in vitro* ICS (IFNγ)	DRB1∗14:04/15:01; DQA1∗01:04/01:02; DGB1∗05:03/06:02P	HLA typing in responding patients	IEDB Consensus tool (ANN+SMM)	
21-40	VNGRKKLEELER	88	1/32	*Ex vivo ELISpo*t	*in vitro* ICS (IFNγ); *ex vivo ELISpo*t IFNγ	DRB1∗10:01	*In silico, HLA t*yping in responding patients	IEDB Consensus tool (ANN+SMM)	Confirmed by *ex vivo ELISpo*t in acutely superinfected patient
31-50	ERDLRKIKKKIKK	88	1/32	ICS	*in vitro* ICS (IFNγ)	DRB1∗10:01	*In silico, HLA t*yping in responding patients	IEDB Consensus tool (ANN+SMM)	
51-70	LGNIKGILGKKDK	88	1/32	ICS	*in vitro* ICS (IFNγ)	DRB1∗15:02	*In silico, HLA t*yping in responding patients	IEDB Consensus tool (ANN+SMM)	
61-80	KDKDGEGAPPA	88	1/32	ICS	*in vitro* ICS (IFNγ)	DRB1∗11:01P; DQA1∗05; DQB1∗03:01P	HLA typing in responding patients	IEDB Consensus tool (ANN+SMM)	
71-90	AKRARTDQMEID	88	2/32	ICS	*in vitro* ICS (IFNγ)	DRB1∗15:01/03:01	*In silico, HLA t*yping in responding patients	IEDB Consensus tool (ANN+SMM)	
81-100	IDSGPRKRPLRG	88	1/32	ICS	*in vitro* ICS (IFNγ)	DRB1∗04:05	*In silico, HLA t*yping in responding patients	IEDB Consensus tool (ANN+SMM)	
111-130	KRKQLAGGGKSL	88	1/32	ICS	*in vitro* ICS (IFNγ)	DRB1∗15:01	*In silico, HLA t*yping in responding patients	IEDB Consensus tool (ANN+SMM)	
141-160	ERRVAGPQVGG	88	1/32	ICS	*in vitro* ICS (IFNγ)	DRB1∗15:01	*In silico, HLA t*yping in responding patients	IEDB Consensus tool (ANN+SMM)	
151-170	GVNPLEGGSRG	88	1/32	ICS	*in vitro* ICS (IFNγ)	DRB1∗11:04/13:03; DQA1∗05; DQB1∗03:01P	HLA typing in responding patients	IEDB Consensus tool (ANN+SMM)	
171-190	MQGVPESPFTRH	88	1/32	ICS	*in vitro* ICS (IFNγ)	DRB1∗11:04/13:03; DQA1∗05; DQB1∗03:01P	HLA typing in responding patients	IEDB Consensus tool (ANN+SMM)	
181-200	RHGEGLGVRGG	88	1/32	ICS	*in vitro* ICS (IFNγ)	DRB1∗11:01	*In silico, HLA t*yping in responding patients	IEDB Consensus tool (ANN+SMM)	

HDAg, hepatitis delta antigen; ICS, intracellular cytokine staining.

In contrast to CD4+ epitopes that are distributed through the entire HDAg (with hotspots at the N-terminus) and can be presented by multiple HLA types, the known HDV-specific CD8+ T cell epitopes seem to cluster in a few distinct locations (mainly C-terminal) and are restricted mainly by HLA-B subtypes, including relatively infrequent subtypes.

A schematic overview of the CD8+ T cell epitopes and their localisation within the HDAg is provided in Fig. 5.

### Viral escape from HDV-specific CD8+ T cell responses

Mutational viral escape is a major mechanism of virus-specific T cell failure in persistent viral infections. Viral escape was first described in the lymphocytic choriomeningitis virus mouse model,^105^ and has been best characterised in HIV and HCV infection in humans.^106^ While there is good evidence that viral escape from virus-specific CD8+ T cell responses impacts the outcome of infection, *e.g.* viral clearance *vs.* persistence, there is less evidence for a role of viral escape from virus-specific CD4+ T cell responses. Mutational escape can either occur at HLA binding anchors of epitopes (mostly aa residue 2 and the C-terminal aa residue), at the T cell receptor contact residues of the epitope (mostly the aa residues in the middle of the epitope), or even in the flanking regions of the epitope, interfering with proteasomal processing of the epitope.^106^ Since HDV has a relatively high mutation rate (see above), it is reasonable to argue that viral escape from virus-specific CD8+ T cell responses may also take place in persistent HDV infection. Indeed, a first study on this issue longitudinally sequenced HDV quasispecies in 4 HLA-A∗02+ patients with chronic HBV/HDV infection before and after hepatitis flares. They found evidence for selection pressure in the HLA-A∗02-restricted epitope aa43-51 KLEDDNPWL in 3/5 patients and in another predicted HLA-A∗02-restricted epitope aa114-122 QLSAGGKSL in 4/5 patients.^107^ However, functional T cell assays to confirm that the patients indeed targeted these epitopes were not performed and experimental evidence for the impact of the observed sequence mutations on recognition by epitope-specific CD8+ T cells or at least HLA-A∗02 binding was not supplied. Similarly, a more recent study performed a cross-sectional sequence analysis in 34 patients with chronic HBV/HDV infection and identified codons 136-159 to be under positive selection pressure, likely indicating CD8+ or B cell pressure.^43^ Unfortunately, HLA typing is not available. The HDAg region under positive selection pressure (aa136-159) overlaps with the HLA-B∗41-restricted epitope aa140-149 RERRVAGPPV, however, this CD8+ T cell epitope (restricted by a rather infrequent HLA class I allele) is unlikely to exert selection pressure at a population level. Due to the dominant role of the HLA class I type B∗27 in driving viral escape in HIV and HCV infection, the HLA-B∗27 background was also used in a pioneer study to functionally demonstrate viral escape from HDV-specific CD8+ T cell responses.^91^ Indeed, 2 predicted HLA-B∗27-restricted epitopes (aa99-108 RRDHRRRKAL and aa103/4-112 (R)RRKALENKK) were identified. Viral sequence analysis in 8 HLA-B∗27+ *vs*. 96 HLA-B∗27- patients demonstrated an enrichment of aa mutations in the epitope region in HLA-B∗27+ patients. These HLA-B∗27-associated viral sequence polymorphisms (also referred to as HLA-B∗27 footprints) indicated that viral escape occurs within these 2 HLA-B∗27-restricted HDV-specific CD8+ T cell epitopes. This was functionally confirmed by intracellular IFN-γ staining using wild-type *vs.* variant peptide, showing little cross-recognition of the variant peptide by wild-type-specific T cell lines.^91^ In a following multicentre, multinational study analysing HLA footprints for all HLA class I alleles present in a cohort of 104 patients with chronic HBV/HDV infection, a total of 21 HLA class I footprints were identified.^92^ Interestingly, these footprints were restricted by relatively infrequent HLA class I alleles, which might indicate that HDV has already adapted to its host’s HLA class I background at a population level, leading to the extinction of HDV-specific CD8+ T cell epitopes restricted by frequent HLA class I alleles. The most striking example of viral escape affected the HLA-B∗15:01-restricted HDV-specific CD8+ T cell epitope aa170-179 SMQGVPESPF: All 8 HLA-B∗15:01+ patients in the cohort displayed a viral sequence mutation at the N-terminal amino acid residue (S170N) that was detected in a minority of HLA-B∗15:01-negative patients only. This mutation impaired cross-recognition by the epitope-specific CD8+ T cell response. The loss of viral control in a patient with acute HDV superinfection coincided with the evolution of this escape mutation, indicating the biological significance of viral escape in HDV persistence. Virus-specific CD8+ T cells targeting this ‘escaped’ epitope displayed a memory-like phenotype (PD-1+CD127+TCF1+) and were thus not terminally exhausted. These data indicate that in parallel to the findings obtained in HCV infection,^108^^,^^109^ viral escape and terminal exhaustion are alternative and non-overlapping mechanisms of virus-specific T cell failure. Unfortunately, it was not possible to analyse the phenotype of HDV-specific CD8+ T cells targeting conserved (‘non-escaped’) epitopes in this study. However, Kefalakes *et al.*^89^ described viral sequence mutations in all 6 HDV-specific CD8+ T cell epitopes identified in 1-4 patients each and could confirm viral escape by HLA binding studies for 6 variant peptides and by functional T cell analysis for 4 variant peptides, respectively. Importantly, HDV-specific CD8+ T cells targeting escaped epitopes displayed a memory-like phenotype (PD-1+CD127+TCF+) without evidence of activation (CD38-), while HDV-specific CD8+ T cells targeting conserved epitopes had a ‘chronically activated’ phenotype (PD-1+CD127^low^TCF1^low^, CD38+). These results further underline the complementary, non-overlapping roles of viral escape and terminal exhaustion in HDV-specific CD8+ T cell failure. In sum, these results highlight an important role of viral escape in HDV persistence and indicate that viral escape needs to be considered in vaccine design.

## Discussion

Forty years after the discovery of HDV, its clinical peculiarities remain enigmatic. Clearly, the fate of the HDV infection is intricately intertwined with the replicative cycle and the clinical course of the HBV infection, since HBsAg loss and HBV seroconversion will ultimately terminate HDV propagation. While the current review focuses on the HDV-specific T cell response, the functional interactions between the 2 viruses and their respective effect on HBV- and HDV-specific adaptive and innate immunity must be studied in much more detail. For example, it is not clear why peg-IFN-α leads to relatively infrequent loss of the HBsAg in patients with HDV compared to those with HBV monoinfection. The immune correlates of spontaneous or therapy-induced control of HDV viraemia – apart from the known fact that HBsAg conversion itself can lead to clearance of HDV – and the role HDV-specific T cell responses play are poorly understood.

It is not clear whether immunological interventions in patients with HDV should a) aim at global blockade of co-inhibitory molecules on T cells, or b) be directly aimed at enhancing the HBV-specific immunity to achieve an HBV seroconversion; it is also not clear whether additionally targeting HDV antigens would synergistically help to achieve this aim. We also do not know whether halting HDV replication on its own – by therapeutic HDV vaccination or antiviral therapy^103^ – would be of significant benefit for chronically HBV/HDV-coinfected patients. Lastly, it is not currently clear whether a prophylactic HDV vaccine would be an epidemiologically useful tool to eradicate HDV infection.^66^^,^^103^

Nonetheless, it is essential to conduct further detailed longitudinal studies on the *ex vivo* phenotype and functionality of the HBV- and HDV-specific T cell response during therapeutic trials to understand the immunological correlates of HDV viral control and to achieve sustained virological responses in the majority of patients with HDV by application of antiviral and immunological combination therapies.

Only some of the recent studies included detailed T cell analysis and only 3 studies utilised *ex vivo* assays like MHC class I multimer stainings. Indeed, due to the generally low *ex vivo* frequencies of circulating HDV-specific T cells,[87], [88], [89] many researchers use peptide pool stimulation, measuring HDV-specific responses after *in vitro* expansion and re-stimulation. Epitope mapping by stimulating T cells with different pools of peptides spanning the whole antigen rather than testing each peptide individually greatly increases efficiency, while assay sensitivity may be slightly reduced, especially when using larger pool sizes.^110^ *In vitro* expansion and subsequent re-stimulation help to identify responses at low frequencies but carry the risk of altered T cell phenotypes and functionalities, limiting the comparability to the *in vivo* situation. Only 3 studies could detect T cell responses directed against 8 different peptide epitopes by direct *ex vivo* staining. Landahl *et al.* detected up to 800 HDV-specific T cells/10^6^ PBMCs by *ex vivo* ELISpot in a patient with acute HDV^88^ and Kefalakes *et al.* performed *ex vivo* multimer stainings in 17 chronically HDV-infected patients after discontinuation of lonafarnib/ritonavir therapy.^89^ Karimzadeh *et al.* managed to detect responses against 2 epitopes *ex vivo* by bead-based CD8+T cell-enrichment, thereby increasing the assay sensitivity^92^ in HLA-matched patients. It is conceivable that a broad and strong specific response is induced in acutely infected patients, which diminishes as the infection persists. Analogously, suppression of viral replication by therapy could lead to partial recovery of exhausted specific memory cells, enabling them to initiate stronger, multi-specific responses upon re-stimulation *ex vivo,* similar to chronic HBV and HCV.^85^

HLA restriction is an additional insufficiently characterised aspect of HDV epitopes. None of the studies describing CD4+ T cell epitopes confirmed the HLA restriction experimentally, *e.g.* using multimer stainings, but rather relied on *in silico* predictions followed by antibody blocking and HLA-haplotype specific co-culturing of CD4+ T cells, or *in vitro* binding assays or HLA fine typing of responding patients. Regarding CD8+ T cell epitopes, 3 studies included multimer assays to confirm HLA restrictions, with 6 epitopes confirmed by direct *ex vivo* multimer staining.^89^^,^^92^

Another aspect which limits the current evidence base is the fact that only 4 studies mapped the whole HDAg for T cell epitopes.[87], [88], [89]^,^^91^ Consequently, only a limited number of patients (8 in Nisini *et al.* 1997, 32 in Landahl *et al.* 2019, 4 in Karimzadeh *et al.* 2018, and 17 in Kefalakes *et al.* 2019) were included in complete mappings. Two studies – Kefalakes *et al.* 2019 and Karimzadeh *et al.* 2018 – focused on CD8+ T cell responses (including 21 patients in total). Landahl *et al.* 2019 aimed to measure both CD8+ and CD4+ T cell responses, although the peptide length of 20 aa is suboptimal for MHC class I presentation, which favour peptides of 8 to 10 aa,[111], [112], [113] and Nisini *et al.* only measured CD4+ responses. Karimzadeh *et al.* mapped HDAg for CD8+ T cell responses by IFN-γ ICS, a high-quality method for epitope mapping, although limited by the small number of patients. In this sense, a particular strength of the study conducted by Kefalakes *et al.* 2019 is the relatively large number of individual patients mapped by a high-quality method (namely ICS). Additionally, the authors were able to confirm epitopes and HLA binding by *ex vivo* multimer stainings of untreated HBV/HDV patients, and even to further characterise HDV-specific CD8+ T cells as discussed earlier in this review. Other studies performed *in silico* predictions of most probable epitopes and their HLA restrictions, which were subsequently experimentally confirmed. This approach carries the inherent risk of missing responses to epitopes with low binding affinities or restriction by uncommon HLA types. Usage of alternative binding pockets in MHC class I molecules and generally shallower (and thus more variable) binding pockets in MHC class II molecules further complicates this approach.^114^ Additionally, CD8+ T cell epitopes may span longer aa sequences than classically assumed, and thus be overlooked by *in silico* predictions presuming lengths of 8 to 10 aa.^111^

Of note, most HDV peptide sets used to experimentally screen for HDV-specific T cell responses are based on single genotype 1-based sequences and there is neither a consensus sequence available nor is there an understanding about the degree of cross-genotype reactivity of these epitopes. Optimally, these peptides should be based on, or compared with, autologous circulating HDV sequences to rule out T cell responses against suboptimal heterologous sequence variants.^115^^,^^116^

Furthermore, studies analysing the breadth, specificity, and functionality of the intrahepatic HDV-specific CD8+ T cell response have not yet been performed.

In summary, we have provided a detailed review of the current knowledge on HDV-specific T cells and a database of all human T cell epitopes of the hepatitis delta virus characterised to date. This evidence base will help to further elucidate the complicated immunology of this enigmatic viral infection that still has grave clinical implications for too many patients.

## Financial support

10.13039/501100001659DFG (German Research Foundation) grants to Julian Schulze zur Wiesch (SFB841 and SFB1328) and Christoph Neumann-Haefelin (TRR-179 “Determinants and dynamics of elimination versus persistence of hepatitis virus infection”, Project 02). 10.13039/100009139German Center for Infection Research (DZIF) grants to Julian Schulze zur Wiesch and Christoph Neumann-Haefelin.

## Authors’ contributions

Conception MK, JL and JSzW; Draft MK, JSzW and CNH; Proofread all authors, important contributions all authors.

## Conflict of Interest

The authors declare that the research was conducted in the absence of any commercial or financial relationships that could be construed as a potential conflict of interest.

Please refer to the accompanying ICMJE disclosure forms for further details.
